# Idiopathic and acquired pedophilia as two distinct disorders: an insight from neuroimaging

**DOI:** 10.1007/s11682-020-00442-z

**Published:** 2021-01-28

**Authors:** Cristina Scarpazza, Livio Finos, Sarah Genon, Laura Masiero, Elena Bortolato, Camilla Cavaliere, Jessica Pezzaioli, Merylin Monaro, Nicolò Navarin, Umberto Battaglia, Pietro Pietrini, Stefano Ferracuti, Giuseppe Sartori, Andrea S. Camperio Ciani

**Affiliations:** 1 Department of General Psychology, University of Padova, Via Venezia 8, 25131 Padova, PD Italy; 2 Department of Psychosis Studies, Institute of Psychiatry, Psychology and Neuroscience, King’s College London, London, UK; 3 Department of Developmental Psychology and Socialisation, University of Padova, Padova, Italy; 4 Institute of Neuroscience and Medicine, Brain and Behaviour (INM-7), Research Centre Jülich, Jülich, Germany; 5 Department of Statistical Sciences, University of Padova, Padova, Italy; 6 Department of Mathematics “Tullio Levi-Civita”, University of Padova, Padova, Italy; 7 Department of Philosophy, Sociology, Education and Applied Psychology, University of Padova, Padova, Italy; 8 Molecular Mind Lab, IMT School for Advanced Studies Lucca, Lucca, Italy; 9 Department of Human Neurosciences, “Sapienza” University of Rome, Rome, Italy

**Keywords:** Idiopathic pedophilia, Acquired pedophilia, Coordinate based meta‐analysis, Lesion network analysis, Behavioral profiling, Neuroimaging

## Abstract

Pedophilia is a disorder of public concern because of its association with child sexual offense and recidivism. Previous neuroimaging studies of potential brain abnormalities underlying pedophilic behavior, either in idiopathic or acquired (i.e., emerging following brain damages) pedophilia, led to inconsistent results. This study sought to explore the neural underpinnings of pedophilic behavior and to determine the extent to which brain alterations may be related to distinct psychopathological features in pedophilia. To this aim, we run a coordinate based meta-analysis on previously published papers reporting whole brain analysis and a lesion network analysis, using brain lesions as seeds in a resting state connectivity analysis. The behavioral profiling approach was applied to link identified regions with the corresponding psychological processes. While no consistent neuroanatomical alterations were identified in idiopathic pedophilia, the current results support that all the lesions causing acquired pedophilia are localized within a shared resting state network that included posterior midlines structures, right inferior temporal gyrus and bilateral orbitofrontal cortex. These regions are associated with action inhibition and social cognition, abilities that are consistently and severely impaired in acquired pedophiles. This study suggests that idiopathic and acquired pedophilia may be two distinct disorders, in line with their distinctive clinical features, including age of onset, reversibility and *modus operandi*. Understanding the neurobiological underpinnings of pedophilic behavior may contribute to a more comprehensive characterization of these individuals on a clinical ground, a pivotal step forward for the development of more efficient therapeutic rehabilitation strategies.

**Supplementary Information:**

The online version contains supplementary material available at 10.1007/s11682-020-00442-z.

## Introduction

Pedophilia is a paraphilic disorder included within the Diagnostic and Statistic Manual of Mental Disorder (fifth edition, American Psychiatric Association 2013) idefined as sexual preferences for prepubescent children, coupled with distress caused by the sexual urges and/or child sexual (American Psychiatric Association 2013; Regier et al. 2013). Although pedophiles are relatively rare (prevalence of 3–5% in the male population (Beech et al. 2016)), they commit a disproportionate amount of crimes and rarely comply with psychological treatments (Hall and Hall 2007). Pedophilia raises high public concern due to its association with child sexual offense and recidivism (Hanson et al. 2003).

Though the multifactorial origin of pedophilia still remains elusive (Mohnke et al. 2014; Tenbergen et al. 2015), recent neuroimaging studies have shown pedophilia to be associated with reduced grey (Poeppl et al. 2013; Schiffer et al. 2007, 2017) and white (Cantor and Blanchard 2012; Cantor et al. 2008, 2015) matter in brain regions involved in sexual arousal (Tenbergen et al. 2015), including amygdala (Poeppl et al. 2013; Schiffer et al. 2007; Schiltz et al. 2007), hypothalamus and septal regions (Poeppl et al. 2013; Schiltz et al. 2007), as well as in the orbitofrontal cortex (OFC) and basal ganglia (Schiltz et al. 2007), areas with a relevant role in impulse inhibition and reward. Pedophiles also showed significant functional activation differences while viewing images depicting nude children and nude adults as compared to controls (Schiffer et al. 2008). Overall, brain imaging studies have revealed a widespread dysfunctional brain activity mainly encompassing the frontal, parietal and temporal lobes (Poeppl et al. 2011; Walter et al. 2007), as well as relevant subcortical structures (Sartorius et al. 2008). However, structural and functional abnormalities in pedophiles show considerable variability across studies. Furthermore, it is unclear to what extent these abnormalities are an incidental correlate rather than a cause of pedophilia (Mohnke et al. 2014; Tenbergen et al. 2015).

Psychiatric symptoms can emerge as a consequence of neurological insult (Keshavan and Kaneko 2013; McAllister 2008); thus, an effective approach commonly adopted by classical neuropsychology, is to investigate the neural basis of pedophilia is to study patients who develop pedophilic urges and/or behavior following focal brain lesions, referred to as “acquired pedophilia” (Camperio Ciani et al. 2019; Gilbert and Focquaert 2015). Unlike “idiopathic pedophilia”, whose etiology remains unknown, acquired pedophilia occurs *de novo* in individuals who had never manifested pedophilic interests or urges earlier in life, as a symptom of an underlying neurological disorder (Devinsky et al. 2010; Fumagalli et al. 2015; Gilbert and Vranic 2015; Gilbert et al. 2016; Mendez and Shapira 2011; Mendez 2010; Mendez et al. 2000; Miller et al. 1986; Sartori et al. 2016; Scarpazza et al. 2018b). The causal inference is strongly indicated by the temporal relationship between the onset of the neurological disorder and the appearance of pedophilic behavior (Scarpazza et al. 2018a). Furthermore, pedophilic behavior recedes after the underlying neurological condition has been treated (Burns and Swerdlow 2003; Sartori et al. 2016). The first documented case of acquired pedophilia, reported in 1862 (von Krafft-Ebing 1897), was a 78 years-old man with no previous criminal record who sexually assaulted a 13 years-old child playing in the garden. Upon medical examination, the subject manifested memory deficits and tangential language and was unable to recognize the moral disvalue or the legal implications of his behavior. Eventually, he was diagnosed with dementia. More recent cases of acquired pedophilia in the literature include: a 40 years-old man with a tumor in the OFC (Burns and Swerdlow 2003); a 67 years-old man with hippocampal sclerosis (Mendez and Shapira 2011); a 50 years-old man with a glioma involving the thalamus, hypothalamus, ventral midbrain an pons (Miller et al. 1986). The above examples already indicate that the mere lesion localization is not enough to account for the neurological bases of acquired pedophilia, as different cases do implicate different brain regions.

Overall, brain imaging studies in idiopathic and acquired pedophilia are inconclusive, as they show subtle and inconsistent brain alterations in developmental pedophilia, and spatially heterogeneous brain lesions in acquired pedophilia. Furthermore, it is not clear whether and to what exent idiopathic and acquired pedophilia may share the same anatomical substrate.

Thus, the current study wished to: (i) identify the brain regions consistently impaired in idiopathic and acquired pedophilia; (ii) determine whether the two forms of pedophilia are associated with overlapping or distinct brain networks; (iii) link topographically defined regions with corresponding psychological processes, testing which kind of experiments are most likely to activate a given region, to give a cognitive/psychological meaning to the detected alterations.

In order to identify brain regions consistently impaired in idiopathic pedophilia, a coordinate based meta-analysis using the Activation Likelihood Estimation (ALE) method was performed (Eickhoff et al. 2012). This approach revealed converging and consistent findings across different studies, underlying important nodes of network alteration in idiopathic pedophilia.

Because in acquired pedophilia only cases reports, with macroscopic neuroanatomical alterations, have been published, the above strategy cannot be adopted. Thus, we used a lesion network mapping approach to identify brain regions consistently impaired in acquired pedophilia (Darby et al. 2018a, b). By assuming that every brain region is a part of complex network, this method identifies regions functionally connected to a lesion (Avena-Koenigsberger et al. 2017). As a matter of fact, lesions causing the same symptoms tend to be functionally connected with the same brain regions (Darby et al. 2018a, b).

## Materials and methods

### Idiopathic pedophilia

#### Study selection and data extraction

An in-depth search was conducted on Pubmed up to January 2020. The following terms were used: (“pedophilia” OR “pedophilic behavior” OR “child* abuse”) AND (“fMRI” OR “functional magnetic resonance imaging” OR “neural basis” OR “voxel based morphometry” OR “brain abnormal*”). A search for studies in review and meta-analysis articles and a reference tracing were also performed.

To be included in the analysis, studies had to meet the following criteria: (i) use structural (sMRI) or functional (fMRI) MRI; (ii) perform a whole brain analysis (i.e., studies performing only region of interest (ROI) analysis were excluded); (iii) be original peer-reviewed data; (iv) include both pedophilic individuals and a healthy control group (HC) or pedophilic individuals who committed and who did not commit sexual abuse; (v) have a sample size of at least five individuals per group; (vi) report results in a standardized coordinate space (e.g., Tailarach Atlas or Montreal Neurologic Institute, MNI).

Literature screening and selection was performed according with the PRISMA guidelines (Moher et al. 2009). Two authors (CS and MM) screened the data independently. A third opinion (UB) was sought in case of discordance. Characteristics such as sample size, age of participants, coordinate space, coordinates and statistical values were recorded.

#### Statistical analysis

For a quantitative assessment of inter-study concordance, the Activation Likelihood Estimation (ALE) method (Eickhoff et al. 2009; Laird et al. 2005; Turkeltaub et al. 2002) was applied to both structural and functional data, following the most recent guidelines (Müller et al. 2018).

The peaks of activation/deactivation or of increased/decreased grey matter volume were used to generate an ALE map, using the revised ALE algorithm (Turkeltaub et al. 2012) running under Ginger ALE software (http://brainmap.org/ale/) version 3.0.2. This algorithm treats activated foci of brain regions as three-dimensional Gaussian probability distributions centered at the given coordinates (Eickhoff et al. 2009; Laird et al. 2005). The algorithm incorporates the size of the probability distributions by considering the sample size of each study. Moreover, it employs the random-effect rather than the fixed-effect inference, by testing the above-chance clustering between contrasts rather than between foci. If the study reported more than one contrast of interest (for instance, brain activation while seeing adult vs. child naked bodies), only the more representative contrast of the process of interest was selected. This procedure was applied to adjust for multiple contrasts from the same sample (Müller et al. 2018). Then, inference was sought regarding the regions in which the likelihood of activation reported in a particular set of experiments was higher than expected by chance. Tailarach coordinates were transformed into MNI using a linear transformation (Laird et al. 2010; Lancaster et al. 2007). Statistical parametric maps were thresholded using a cluster level family-wise error (FWE) correction at p < 0.05 (cluster-forming threshold at voxel-level p < 0.001 (Eickhoff et al. 2016)). For explorative analyses only, a p < 0.001 uncorrected threshold was used. The correspondent brain regions were identified using the SPM Anatomy toolbox (version 1.5) (Eickhoff et al. 2005). For further details on the ALE method please refer to previous publications (Eickhoff et al. 2009, 2012b; Turkeltaub et al. 2012).

### Acquired pedophilia

#### Study selection and data extraction

Published cases of acquired pedophilia were identified through a systematic review (Camperio Ciani et al. 2019). To be included in the analysis, studies had to: (i) be original reports of late onset pedophilic behavior; (ii) report a documented neurological condition temporally associated with the emergence of the pedophilic behavior; (iii) have a clearly identifiable neural basis for the pedophilic behavior. Two authors (CS and UB) extracted and screened the data independently. A third opinion (MM) was sought in case of discordance. Information as age of the offender, etiology of the underlying neurological disorder, brain localization of neuroanatomical abnormalities and symptoms other than pedophilia were recorded.

### Statistical analysis

The lesion network mapping analysis (Darby et al. 2018a, b) was run to determine whether these lesion locations were part of a common brain network.

First, the brain alterations associated with the onset of pedophilic behavior were identified in each individual patient and manually traced in consensus by two expert raters (CS and UB) on the axial image of a standardized template using the MRIcron software (available at http://www.mricro.com/mricron) (Rorden and Brett 2000; Rorden et al. 2009). Then, the lesion outline was verified by an independent third rater (SF). Some of the patients presenting with *de novo* pedophilia were diagnosed with a behavioral variant of frontotemporal dementia (bvFTD); therefore, they did not present a spatially defined lesion that could be outlined. In order to identify the neural structures consistently impaired in bvFTD, a coordinate based meta-analysis was run on papers presenting structural or functional abnormalities in patients with bvFTD vs. healthy controls (see Supplementary Material A and B for details). The output of the meta-analysis was then transformed in a binary mask.

Second, traced lesions were used as individual seeds in a seed based connectivity analysis, using resting state fMRI data from one hundred healthy subjects randomly selected from a freely available dataset: https://openneuro.org/datasets/ds000221. The brain functional connectivity with each lesion was determined by calculating the correlated time course between each lesion location and every other brain voxel using the resting-state data from each individual healthy control, as previously reported (Darby et al. 2018a, b). The results in all controls were combined into an average correlation (r), converted according to Fisher transformation (z) using the following formula:

$$z=\frac{1}{2}log(\frac{1+r}{1-r})=arctanh(r)$$

and then modeled in a linear regression framework to obtain a T value for each individual voxel and each brain mask. Voxels were thresholded at T > ± 17 to create a binarized map of significantly functionally connected regions to the seed, that corresponded to an effect size of R^2^ = 0.75. An extent threshold of 50 voxels also was applied. In this way, the brain network impaired by the presence of each lesion was calculated. Finally, maps from each of the patients were combined to form the lesion network mapping overlap for the group, showing the number (and percentage) of patients with lesions functionally connected to each individual voxel. A stability analysis was performed by replicating the analyses using three different control groups, each with 25 healthy subjects (we kept the minimum effect size of R^2^ = 0.75, which implies a T > ± 8.5). Analyses were performed using SPM-CONN (2018b) adopting standard preprocessing and denoising steps.

### Behavioral profile

To link topographically defined brain regions with the corresponding psychological process, we ran a behavioral profiling approach across databases of aggregation from activations experiments (Genon et al. 2018; Plachti et al. 2019). This approach identifies which kind of experiments are most likely to activate a given region. A reverse inference approach with statistical testing (P < 0.05 corrected for multiple comparisons) was performed on the identified clusters of voxels in BrainMap database (http://www.brainmap.org/), to reveal the Behavioral Domain and Paradigm Classes consistently associated with these regions.

## Results

### Idiopathic pedophilia

One hundred and eighty studies were identified. After excluding the papers that did not meet the inclusion criteria, 19 original articles were included. The screening procedure, summarized in the PRISMA diagram, and the reasons for excluding individual studies are reported in the Supplementary Material C.

The included studies are summarized in Table 1 and the full database reporting the coordinates is available in the Supplementary Material D. Briefly, the coordinate based meta-analysis comprises 20 experiments (one study (Kargel et al. 2015) included two independent groups of pedophiles), 240 foci, 436 pedophiles and 449 control individuals, of whom 302 were healthy controls, 50 were non sexual offenders, and 97 were pedophiles who did not commit sexual offenses toward children. Critically, the included studies were not completely independent as some came from the same laboratories and at least a partial sample overlap was reported in some studies.

**Table 1:** Characteristics of the studies included in the ALE meta-analysis on idiopathic pedophilia

	Reference	Ped. N	Contr. N	Neuroimaging technique	Contrast	Number of foci
1	(Schiffer et al. 2007)	18	24 HC	sMRI	Pedophiles vs. HC	20
2	(Walter et al. 2007)	13	13 HC	fMRI	Pedophiles vs. HC	15
3	(Sartorius et al. 2008)	10	10 HC	fMRI	Pedophiles vs. HC	2
4	(Schiffer et al. 2008)	8	12 HC	fMRI	Pedophiles vs. HC	32
5	(Schiffer et al. 2008)	11	12 HC	fMRI	Pedophiles vs. HC	9
6	(Poeppl et al. 2011)	9	11NSO	fMRI	Pedophiles vs. NSO	13
7	(Ponseti et al. 2012)	24	18 HC	fMRI	Pedophiles vs. HC	25
8	(Habermeyer et al. 2013a)	11	7 HC	fMRI	Pedophiles vs. HC	4
9	(Habermeyer et al. 2013b)	8	8 HC	fMRI	Pedophiles vs. HC	12
10	(Poeppl et al. 2013)	9	11 NSO	sMRI	Pedophiles vs. NSO	10
11	(Cantor et al. 2015)	24	32 HC	sMRI	Pedophiles vs. HC	30
12	(Gerwinn et al. 2015)	24	32 HC	sMRI	Pedophiles vs. HC	3
13	(Kargel et al. 2015)	12 CSA+	14 HC	fMRI	Pedophiles vs. HC	13
		14 CSA-	14 HC	fMRI	Pedophiles vs. HC	3
14	(Cantor et al. 2016)	37	39 HC + 28 NSO	fMRI	Pedophiles vs. (HC + NSO)	23
15	(Kargel et al. 2017)	40 CSA+	37 CSA-	fMRI	CSA + vs. CSA-	3
16	(Massau et al. 2017)	31	19 HC	fMRI	Pedophiles vs. HC	4
17	(Schiffer et al. 2017)	58 CSA+	60 CSA-	fMRI	CSA + vs. CSA-	8
18	(Ponseti et al. 2017)	60	55 HC	fMRI	Pedophiles vs. HC	9
19	(Fonteille et al. 2019)	15	15 HC	PET	Pedophiles vs. HC	2

Ped. N = Number of pedophiles; Contr. N = number of controls; HC = Healthy Controls; NSO = Non Sexual Offenders; CSA + = pedophiles who committed child sexual abuse; CSA- = pedophiles who did not commit child sexual abuse; sMRI = structural magnetic resonance images; fMRI = functional magnetic resonance images

**Fig. 1:**
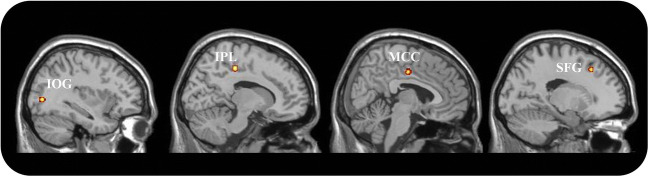
Results of ALE-meta-analysis in idiopathic pedophilia. Results are presented in the sagittal view for illustrative purposes only at the liberal statistical threshold of *p* < 0.001, uncorrected. IPL: Inferior Parietal Lobe; MCC: Middle Cingulate Cortex; IOG: Inferior Occipital Gyrus; SFG = Superior Frontal Gyrus

Using a conservative statistical threshold, no significant results were found, though the power of the analysis would have been sufficient to achieve significant results (Eickhoff et al. 2016). For exploratory purposes only, the threshold was decreased to p < 0.001 uncorrected. Using this liberal threshold, four clusters located in the middle occipital gyrus (coordinates: -36, -78, 2), in the middle cingulate gyrus (coordinates: 8, -12, 42 and 12, -30, 46) and in the superior frontal gyrus (coordinates: -17, 24, 45) were detected (Fig. 1). Although the whole meta-analysis included some studies with partially overlapping samples, these studies did not contribute to the creation of significant clusters. Thus, the foci contributing to each cluster came from independent samples. For this reason, and given the exploratory nature of this second analysis, the meta-analysis was not repeated after removing the partially overlapping samples. The behavioral profiling analysis was not performed in order to avoid over-interpretation of statistically non-significant results.

### Acquired pedophilia

Seventeen papers were identified through the literature search, for a total of nineteen cases that met the inclusion criteria (see Supplementary Material E for details on the excluded cases). Table 2 reports the age, etiology, neural basis and symptoms and signs presented by each patient.

**Table 2:** Clinical characteristics of the patients with acquired pedophilia

Reference	Age	Neural basis	Etiology	Medication	Symptoms
(Lesniak et al. 1972)	60	Right Frontal lobe	Tumor (benign glioma)	Not reported	Coprolalia, exhibitionism, quick tempered and irritable, impairment of smell, hypersexuality
(Regestein and Reich 1978) Case 1	56	OFC	Supracellar meningioma	Not reported	Decreased vision in the left eye, right side facial weakness, hyperreflexia, personality change, lack of initiative, impaired moral reasoning, impaired prosody, absence of insight
(Miller et al. 1986) Case 5	50	Left brainstem, hypothalamus, thalamus	Hypercellular grade 3 astrocytoma	Not reported	Subtle personality changes, poor financial and moral judgement, hemiparesis, hemiataxia
(Mendez et al. 2000) Case 1, Also described in (Mendez and Shapira 2011; Mendez 2010) Case 1	60	Atrophy in the frontal and temporal cortices	bvFTD	Paroxetine; valproate; estrogens	Decline in social and personal awareness, dis-inhibition, hyperorality, lack of insight, utilization behavior, echolalia, verbal stereotypies, impaired memory, lack of abstract thinking, compulsive behaviours
(Mendez et al. 2000) Case 2, also described in (Mendez and Shapira 2011) case 8	67	Bilateral hyppocampi	Hippocampal sclerosis	Sertraline (history of cocaine abuse)	Severe memory difficulties, 24/30 at MMSE, normal language abilities
(Frohman et al. 2002)	38	Hypothalamus, brainstem, right sub-insula regions, basal ganglia	Multiple Sclerosis	Interferon beta-1b (for multiple sclerosis); fluvoxamine maleate; medroxyprogesterone acetate	Binocular diplopia, dysarthria, ataxia, poor judgement, impulsivity, dis-inhibition, perseveration, hypersexuality
(Burns and Swerdlow 2003)	40	Right OFC	Hemangio-pericytoma	Fluoxetine hydrochloride, amlodipine besylate, metoclopramide hydrochloride, medroxyprogesterone acetate	Dis-inhibition, spared moral reasoning, constructional apraxia, writing illegible, balance problems, incontinence
(Devinsky et al. 2010)	51	Right mesial temporal lobe	Gangloglioma	Antiepileptic drugs; quetiapine and sertraline (after the arrest)	Musical hallucinations, personality changes, irritability, dis-inhibition (manifesting with Kluver-Bucy symptoms of hyperphagia, coprophilia), hypersexuality
(Rainero et al. 2011)	49	Bilateral frontal lobe atrophy	bvFTD	Not reported	Deficit in episodic memory, verbal aggressiveness, severe impairment in frontal functions as revealed by the neuropsychological assessment, social detachment, reduced insight and disease awareness
(Mendez and Shapira 2011) Case 2	67	Bilateral frontal lobe atrophy	bvFTD	Haloperidol	Insidious Personality change, lack of insight, dis-inhibition, compulsive acts, hyperorality, decreased verbal fluency, hypersexuality
(Mendez and Shapira 2011) Case 4	82	Right globus pallidus	Vascular dementia	Valproate; trazodone	Sudden onset of personality changes, dis-inhibition, baby talking, profane language, perseveration, stimulus-bound behavior, hypersexuality
(Mendez and Shapira 2011) Case 6	32	Caudate, putamen and striatum bilaterally	Huntington’s disease	Haloperidol; Sertraline	Personality changes, dysartria, aggressiveness, decreased verbal fluency, deficit in executive functions, lack of insight, impulsivity
(Mendez and Shapira 2011) Case 7	59	Right Pallidum	Right Pallidotomy	Carbidopa/levodopa; pramipexole	Spared insight of behavior, dis-inhibition, hypersexuality
(Fumagalli et al. 2015)	63	Right vmPFC, left PFC	TBI	Irbesartan for hypertension; paroxetine	Irritability, uncontrollable emotional reactions, mild dis-inhibition, dysexecutive syndrome, impulsivity
(Gilbert and Vranic 2015; Gilbert et al. 2016)	48	Left frontal lobe	Glioblastoma multiforme	Levetiracetam (Antiepileptic drugs); diazepam;	Epilepsy, depression, apatia, aggressiveness, confusion, dis-orientation
(Alnemari et al. 2016)	Early 20	Basal frontal and bilateral temporal	Epidural hematoma from TBI	Not reported	Attention deficit, difficulty sleeping, irritability, and unspecified behavioral changes
(Sartori et al. 2016; Scarpazza et al. 2018)	64	OFC + Hypothalamus	Clivus Chordoma	Not reported	Dis-inhibition, deficit social cognition, deficit emotion attribution, deficit in understanding morality, anoso-agnosia
(Scarpazza et al. 2018) Case 1	70	Bilateral frontal lobe atrophy	bvFTD	Anti-dopaminergic drugs	Deficit in critical thinking, abstract thinking, severe deficit in attention, behavioral control, impulse inhibition, preservative behavior and an inability to foresee the consequences of his own actions, hypersexuality
(Scarpazza et al. 2018) Case 2	60	Frontal and parietal lobes	Meningothelial Meningioma	Delorazepam	Constructional apraxia, impaired sustained attention, difficulty to inhibit the automatic answer and behavior; impairment in problem solving and planning abilities, perseveration.

OFC = OrbitoFrontal Cortex; vmPFC = VentroMedial Prefrontal Cortex; PFC = Prefrontal Cortex; bvFTD = behavioral variant Fronto Temporal Dementia; TBI = Traumatic Brain Injury

Seven out of the 19 patients expressed hyper-sexuality and all of them manifested a more general impulse dis-control. Moral judgment/social cognition behavior (namely, the ability to understand the social and moral disvalue of their action, theory of mind, ability to discriminate right from wrong) was impaired in nine patients, spared in four, while no data were available for the remaining six cases.

Lesion localization was very heterogeneous, as reported in Table 2. Lesions were traced using the original anatomical scans of the patients in two cases (Sartori et al. 2016; Scarpazza et al. 2019); the images reported in the original publications in six cases (Alnemari et al. 2016; Burns and Swerdlow 2003; Frohman et al. 2002; Fumagalli et al. 2015; Gilbert and Vranic 2015; Gilbert et al. 2016; Mendez et al. 2000); from a coordinate based meta-analysis on bvFTD in four cases Mendez and Shapira 2011; Mendez et al. 2000; Rainero et al. 2011; Scarpazza et al. 2018). In the remaining cases, lesions were traced following the description provided in the paper (Devinsky et al. 2010; Lesniak et al. 1972; Mendez and Shapira 2011; Miller et al. 1986; Regestein and Reich 1978) and following indications from expert neuroradiologists and neurosurgeons when needed. In one case (Devinsky et al. 2010), the author of the original publication verified the traced lesion.

**Fig. 2:**
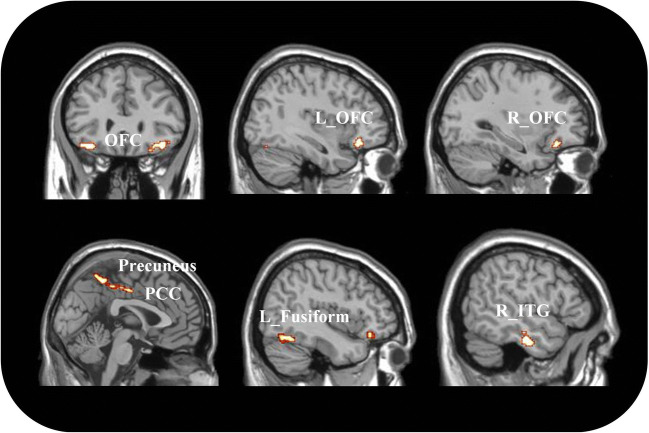
Brain regions consistently involved in acquired pedophilia. OFC = OrbitoFrontal Cortex, PCC = Posterior Cingulate Cortex ; ITG = Inferior Temporal Gyrus; R = right; L = left

Though the individual lesions had different locations, the lesion network mapping analysis revealed that 95% of them were part of a single brain network defined by functional connectivity with posterior midline structures (center of gravity coordinates: 0, -43, 55), including the posterior cingulate cortex (PCC) and precuneus; the bilateral OFC (left coordinates: -34, 32, -17; right coordinates: 36, 30, -17)); the right inferior temporal gyrus (ITG; coordinates: 52, -16, -28), the left calcarine gyrus (coordinates: -9, -56, 7) and the left fusiform gyrys (coordinates: -44, -63, -19) (Fig. 2). These results were replicated using also smaller control groups.

Interestingly, the behavioral profiling analysis (Table 3) highlighted that the identified regions are associated with social cognition (posterior midline structures and inferior temporal gyrus) and in particular with the theory of mind construct (posterior midline structures). Furthermore, a significant association was found between the right OFC and different functions covering emotions and action inhibition. Additionally, regions in the left hemisphere were associated with functions supporting object identification/interpretation, monitoring of information/discrimination judgments and autobiographical remembering.

**Table 3:** Results of the behavioral profiling analysis

Cluster	Size (k)	BrainMap Behavioral Domains	BrainMap Paradigm Classes
Posterior midline regions (precuneus, PCC)	131	Social Cognition	Theory of mind
Left OFC	122	Language Cognition: Semantics, Gustation	Semantic Monitor/discrimination
Right OFC	62	Emotion, Gustation, Action Inhibition	ns
Right ITG	96	Social Cognition	ns
Left Fusiform gyrus	95	Language Cognition: Semantics, Speech, Phonology. Action execution: Speech, Action observation	Face; monitor/discrimination; Phonological discrimination; film viewing; naming (overt); naming (covert)
Left Calcarine Gyrus	55	Explicit (long-term) memory	Autobiographical recall

PCC = Posterior Cingulate Cortex; OFC = OrbitoFrontal Cortex; IFG = Inferior Temporal Gysus; ns = non significant results

## Discussion

This study sought to: (i) identify consistent alterations associated with acquired and idiopathic pedophilia; (ii) understand whether and to what extent the two forms of pedophilia may share the same biological substrate; (iii) investigate whether consistent brain abnormalities may explain psychopathological features typically detected in pedophiles.

Of relevance, the lesion network mapping technique revealed that the neural bases of acquired pedophilia localize to a common resting state network, despite the high spatial heterogeneity of the individual lesions. Overall, these data support a shared neurobiological substrate in acquired pedophilia, as they reveal that the lesions chronologically associated with acquired pedophilic behavior are all functionally connected with a network involving the OFC areas, the posterior midline structures, the right inferior temporal gyrus and the left fusiform gyrus.

On the contrary, the ALE meta-analysis of whole brain neuroimaging studies in idiopathic pedophilia revealed no spatially convergent findings across studies, suggesting that idiopathic pedophilia does not have consistent brain alterations that may be detected by structural or functional neuroimaging investigations. However, when lowering the statistical threshold, a few clusters of spatial convergence emerged in the superior frontal gyrus, middle cingulate and middle occipital gyrus. The different findings obtained from the analyses in idiopathic and acquired pedophilia may suggest that the two conditions may not rely on a shared neural base. Of note, the amygdala, which had been reported to be consistently impaired in pedophilia (Mohnke et al. 2014; Tenbergen et al. 2015), did not emerge from the current meta-analysis, even at the more liberal statistical threshold. This suggests that ROI analyses in original studies may have overestimated the real amygdala effects.

Thus, despite idiopathic and acquired pedophilia are usually considered as a whole in studies that investigate the neural basis of pedophilic behavior, they actually seem to be two distinct disorders. Indeed, they differ in their etiology: while idiopathic pedophilia is a paraphilia, namely a psychiatric disorder included within the DSM5, acquired pedophilia is a symptom resulting from an underlying neurological insult. Second, *modus operandi* in the two conditions widely differs: while idiopathic pedophiles are characterized by a highly predatory style (Fagan et al. 2002; Hall and Hall 2007), acquired pedophiles usually show a dis-organized behavior, characterized by dis-control of impulses (Camperio Ciani et al. 2019; Scarpazza et al. 2018). Third, the temporal insurgence of the pedophilic urges is an additional factor that contributes to the differential diagnosis: while idiopathic pedophilia typically first appears in adolescence and is stable across the lifespan(Beech et al. 2016), the age of the onset of acquired pedophilia is typically well after adolescence and varies depending on the time of onset of the underlying neurological lesion (Camperio Ciani et al. 2019).

The current results provide further support to the existence of two distinct neural networks involved in the two forms of pedophilia, corroborating the emerging idea that they might be two different disorders/ entities(Camperio Ciani et al. 2019).

Interestingly, the data driven behavioral profiling on acquired pedophilia indicated impaired connectivity between lesions causing acquired pedophilia and regions crucial for social cognition (posterior midline structures and right ITG), specifically theory of mind (posterior midline structures), emotion recognition (right OFC), impulse control (right OFC), semantic interpretation of cues (left OFC, L fusiform gyrus). It is noteworthy that these results match well with the aberrant behavior pattern described in acquired pedophiles. The observation of altered activity in a key region for impulse inhibition fits perfectly with previous evidence from single case description of patients with acquired pedophilia, in whom dis-inhibition is invariably present (Devinsky et al. 2010; Gilbert and Focquaert 2015; Mendez and Shapira 2011; Miller et al. 1986; Sartori et al. 2016; Scarpazza et al. 2018). Dis-inhibition also was recently reported to be a red flag suggesting an acquired origin of pedophilic behavior (Camperio Ciani et al. 2019) and accounts for the dis-organized *modus operandi* of these sexual offenders. Similarly, the observation of altered activity in key regions for social cognition, in particular for theory of mind and emotion recognition, fits well with the patient inability to understand what is morally wrong (Camperio Ciani et al. 2019; Frohman et al. 2002; Fumagalli et al. 2015; Mendez and Shapira 2011; Miller et al. 1986; Regestein and Reich 1978; Sartori et al. 2016; Scarpazza et al. 2018).

Of note, these results are specific for acquired pedophilia, as idiopathic pedophilia was not associated with impairments in the same brain regions, even when the statistical threshold was lowered. Idiopathic pedophiles are characterized by a different profile of neuropsychological impairment, consisting in lower IQ and working memory performance, coupled with a higher performance in abstract reasoning and planning, as compared to non pedophiles (Tenbergen et al. 2015). Although difficulties in behavioral inhibition and empathy have also been observed in idiopathic pedophiles, the reported effect size is very small (Tenbergen et al. 2015), suggesting that individual inferences in idiopathic pedophilia are relevant. Furthermore, idiopathic pedophilia has a high comorbidity with other psychiatric disorders, in particular with personality disorders (Fagan et al. 2002; Geer et al. 2000; Kruger and Schiffer 2011; Raymond et al. 1999). Thus, it is difficult to disentangle to what extent the reported neuropsychological impairments are related to pedophilia itself or to the associated psychiatric condition.

Importantly, cognitive abilities associated with brain regions that are impaired in acquired pedophiles comprise impulse control, emotion recognition and social cognition/theory of mind, all functions that are pivotal for self-determination. Indeed, according to the neuroscientific INUS (Insufficient but Non-redundant parts of Unnecessary but Sufficient conditions) model of causation (Anckarsäter et al. 2009), the concomitant impairment in impulse control and social cognition would account for the acquired pedophilic behavior. According to this model, while none of these impaired functions taken by itself in isolation could explain the insurgence of pedophilic behavior, all together they can.

Acquired pedophilia may be considered as a behavioral manifestation of pre-existent latent pedophilic urges due to general impulse dis-inhibition (Mohnke et al. 2014). Interestingly, the application to acquired pedophilia of the INUS model of causation, which requires the concomitant impairment in both social cognition and action inhibition, makes this hypothesis less likely. This claim, however, needs further exploration.

As a final note, the result that idiopathic and acquired pedophilia are characterized by distinct neural networks highlights the need to reconsider the approach of using neurological disorders to investigate the basis of psychiatric conditions or complex behaviors (Darby et al. 2018a). Indeed, individual psychiatric symptoms that may appear within the clinical picture of a neurological condition, like hallucinations or thought disorders in patients with epilepsy or brain tumors, not necessarily may have a neural substrate identical to the one underlying their manifestation in the course of a psychiatric disorder. As a matter of fact, psychiatric and neurological disorders have been proven to have distinct neuroimaging correlates that arguably may reflect distinct neuropathologies (Crossley et al. 2015).

This study is not free from drawbacks. Specifically, some of the seeds of the lesion network analysis were traced without a 2D figure from the original paper so that consultation with neuro-radiologists was necessary to identify the most likely lesion(s). Results of neuroimaging analyses, however, strongly reflect cognitive/behavioral deficits observed in those patients, corroborating the plausibility of our analysis. Second, the lesion network analysis was run using only a relatively small sample of healthy controls (one hundred subjects). The additional analyses we run, however, corroborated the robustness of the results, which remained stable using different control groups of 25 healthy controls. Finally, in the lesion network mapping analysis we could not take into account potential medication effects in the individual patients. However, the original papers indicate that drugs were usually administered after symptoms insurgence, thus the impact of pharmacotherapy for the purposes of the current study is limited. Future studies should assess potential effects of pharmacotherapy.

In summary, the results of this study pinpoint aberrant brain activity related to acquired but not to idiopathic pedophilia. All the lesions causing acquired pedophilia localized to a shared resting state network including the posterior midlines structures, the right inferior temporal gyrus and the bilateral OFC, regions consistently involved in social cognition, theory of mind, emotion recognition and action inhibition. Alterations of these neuropsychological functions have been consistently described in individual reports of acquired pedophiles, in line with the observed results. Interpreting these findings in light of the INUS model of causation is relevant to better characterize these patients and to develop novel therapeutic and rehabilitative strategies (McGorry et al. 2014). Further researches in larger samples are needed to corroborate these results.

## Supplementary Information

ESM 1 (DOC 437 KB)

ESM 2 (PDF 370 KB)

ESM 3 (DOC 92.5 KB)

ESM 4 (PDF 486 KB)

ESM 5 (DOC 47.0 KB)
