# Supplementary material for: Idiopathic and acquired pedophilia as two distinct disorders: an insight from neuroimaging
Source: Brain Imaging Behav. 2021 Jan 28;15(5):2681–92. doi: 10.1007/s11682-020-00442-z (PMC8500885; doi:10.1007/s11682-020-00442-z)
Supplement: Supplementary file 1 — (DOC 437 KB) [file 11682_2020_442_MOESM1_ESM.doc]

**Behavioral Variant Fronto temporal dementia meta-analysis**

Five patients described within the literature manifested acquired pedophilia as a symptom of the behavioral variant of frontotemporal dementia (bvFTD). In order to identify brain regions consistently affected in this neurological disorder, a coordinate based meta-analysis using the ALE algorithm described in the main paper has been performed.

A systematic search of the literature was performed in August 2019. Studies meeting the following inclusion criteria were included in the meta-analysis: i) studies using structural (sMRI) or functional (fMRI) MRI; ii) studies reporting results from a whole brain analysis (i.e. studies performing region of interest (ROI) analysis only were excluded); iii) original peer-reviewed studies and those reporting novel data; iv) studies investigating neural differences between patients with behavioral variant fronto-temporal dementia and healthy controls; v) studies with a sample size >=5 (per group); vi) studies reporting results in a standardized coordinate space (e.g. Tailarach or Montreal Neurologic Institute).

The systematic search revealed 23 studies meeting the inclusion criteria (Agosta et al., 2009; Baez et al., 2016; Baez et al., 2019; Bertoux et al., 2018; Buhour et al., 2017; Hornberger, Geng, & Hodges, 2011; Hua et al., 2018; Irish, Devenney, et al., 2013; Irish, Hodges, & Piguet, 2013; Irish, Piguet, Hodges, & Hornberger, 2014; Kipps, Nestor, Acosta-Cabronero, Arnold, & Hodges, 2009; Lagarde et al., 2015; Lagarde et al., 2013; Lee et al., 2017; Libon et al., 2009; Mandelli et al., 2016; Ossenkoppele et al., 2015; Pardini, Huey, Cavanagh, & Grafman, 2009; Rankin et al., 2011; Rosen et al., 2002; Seeley et al., 2008; Whitwell et al., 2011; Wong et al., 2016). Notably, these studies are more than the one included in a recent coordinate based meta-analysis on bvFTD (Luo et al., 2020). Data necessary to run ALE meta-analysis (reference; number of participants; coordinate system; coordinates) were recorded. ALE meta-analysis was performed using Ginger ALE software (<http://brainmap.org/ale/>) version 3.0.2 as described in the main paper. Statistical parametric maps were thresholded using cluster level family-wise error (FWE) correction at p<0.05 (cluster-forming threshold at voxel-level p<0.001, as suggested by meta-analysis guidelines (Muller et al., 2018).

The bvFTD included 26 experiments (one paper (Seeley et al., 2008) presented data of three independent groups, one paper (Irish, Devenney, et al., 2013) presented data of two independent groups), for a total of 376 foci and 522 patients with bvFTD.

Significant results are reported in the following table and graphically represented in the following Figure.

| **Coordinates** | **Brain region** |
| --- | --- |
| -34 20 4 | Insula |
| -38 16 -12 | Insula |
| 2 36 18 | ACC |
| 38 20 4 | Insula |
| 40 18 -10 | Insula |
| 10 14 8 | Caudate |
| 0 36 38 | MeFG |
| 4 30 44 | MeFG |
| -28 62 -2 | SFG |
| -8 14 8 | Caudate |
| 46 34 24 | MFG |
| -44 12 28 | IFG |
| 3814 32 | Precentral Gyrus |
| -2 54 -4 | ACC |
| -4 10 -6 | Caudate |
| 4 8 -6 | Caudate |
| -30 38 -14 | MFG |
| 30 -14 -14 | Parahippocampus |
| 34 -8 -22 | Parahippocampus |
| -42 52 8 | MFG |
| 2 -8 8 | Thalamus |
| -22 6 6 | Lentiform Nucleus |
| -2 40 -26 | MeFG |
| -54 -30 -6 | MTG |
| 28 6 8 | Lentiform Nucleus |
| 48 -18 -6 | Insula |
| -54 6 -26 | MTG |
| -26 52 18 | SFG |
| 46 -6 32 | Precentral Gyrus |

**Table**. Coordinates and correspondent brain regions emerged as result of the coordinate based meta-analysis on bvFTD. ACC = Anterior Cingulate Cortex; MeFG = Medial Frontal Gyrus; SFG = Superior Frontal Gyrus; MFG = Middle Frontal Gyrus; IFG = Inferior Frontal Gyrus; MTG = Middle Temporal Gyrus.


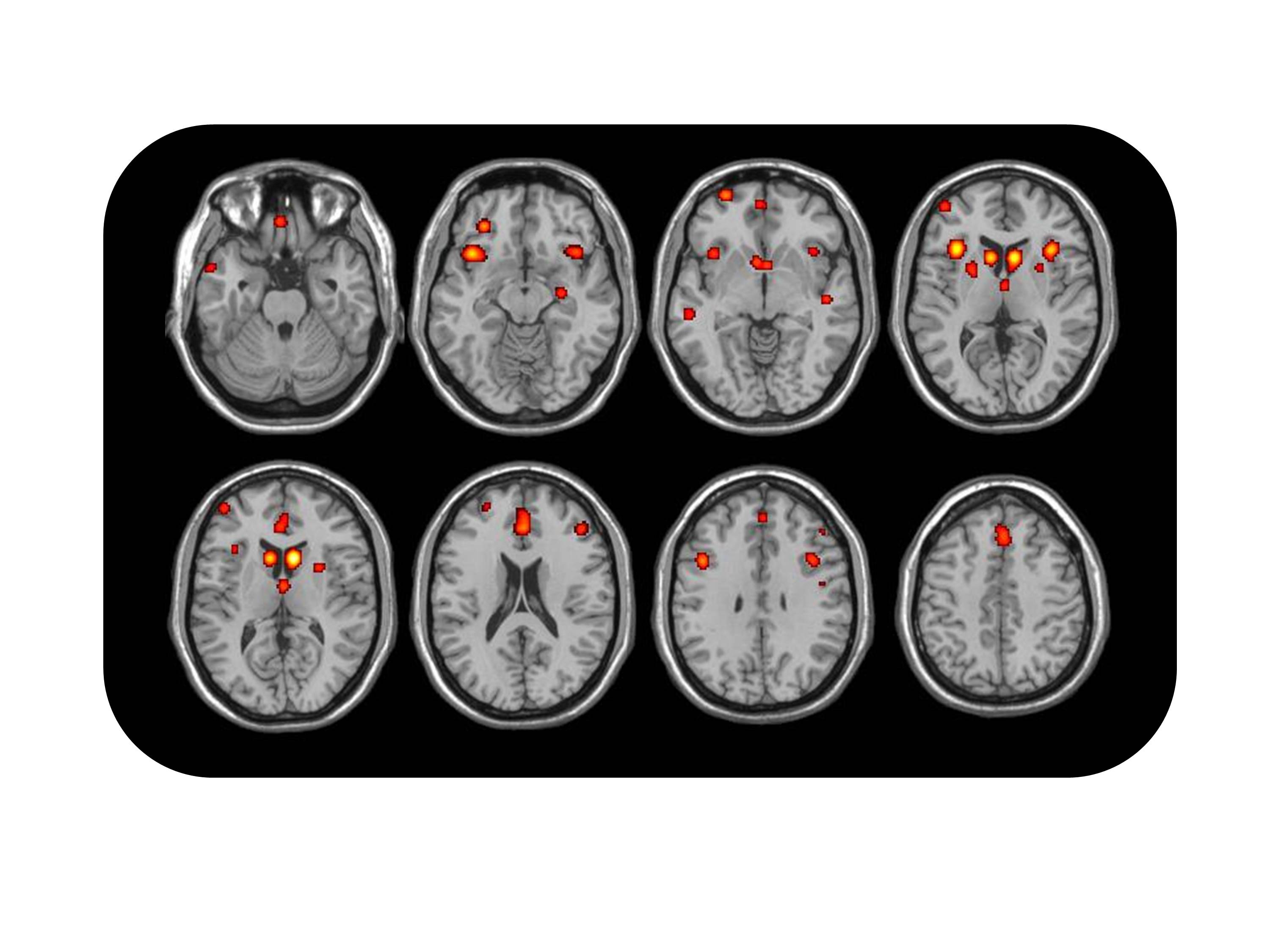


**Figure.** The figure depicted the bvFTD consistent brain alterations

**References**.

Agosta, F., Vossel, K. A., Miller, B. L., Migliaccio, R., Bonasera, S. J., Filippi, M., . . . Gorno-Tempini, M. L. (2009). Apolipoprotein E epsilon4 is associated with disease-specific effects on brain atrophy in Alzheimer's disease and frontotemporal dementia. *Proc Natl Acad Sci U S A, 106*(6), 2018-2022. doi:10.1073/pnas.0812697106

Baez, S., Morales, J. P., Slachevsky, A., Torralva, T., Matus, C., Manes, F., & Ibanez, A. (2016). Orbitofrontal and limbic signatures of empathic concern and intentional harm in the behavioral variant frontotemporal dementia. *Cortex, 75*, 20-32. doi:10.1016/j.cortex.2015.11.007

Baez, S., Pinasco, C., Roca, M., Ferrari, J., Couto, B., Garcia-Cordero, I., . . . Torralva, T. (2019). Brain structural correlates of executive and social cognition profiles in behavioral variant frontotemporal dementia and elderly bipolar disorder. *Neuropsychologia, 126*, 159-169. doi:10.1016/j.neuropsychologia.2017.02.012

Bertoux, M., Flanagan, E. C., Hobbs, M., Ruiz-Tagle, A., Delgado, C., Miranda, M., . . . Hornberger, M. (2018). Structural Anatomical Investigation of Long-Term Memory Deficit in Behavioral Frontotemporal Dementia. *J Alzheimers Dis, 62*(4), 1887-1900. doi:10.3233/JAD-170771

Buhour, M. S., Doidy, F., Laisney, M., Pitel, A. L., de La Sayette, V., Viader, F., . . . Desgranges, B. (2017). Pathophysiology of the behavioral variant of frontotemporal lobar degeneration: A study combining MRI and FDG-PET. *Brain Imaging Behav, 11*(1), 240-252. doi:10.1007/s11682-016-9521-x

Hornberger, M., Geng, J., & Hodges, J. R. (2011). Convergent grey and white matter evidence of orbitofrontal cortex changes related to disinhibition in behavioural variant frontotemporal dementia. *Brain, 134*(Pt 9), 2502-2512. doi:10.1093/brain/awr173

Hua, A. Y., Sible, I. J., Perry, D. C., Rankin, K. P., Kramer, J. H., Miller, B. L., . . . Sturm, V. E. (2018). Enhanced Positive Emotional Reactivity Undermines Empathy in Behavioral Variant Frontotemporal Dementia. *Front Neurol, 9*, 402. doi:10.3389/fneur.2018.00402

Irish, M., Devenney, E., Wong, S., Dobson-Stone, C., Kwok, J. B., Piguet, O., . . . Hornberger, M. (2013). Neural substrates of episodic memory dysfunction in behavioural variant frontotemporal dementia with and without C9ORF72 expansions. *Neuroimage Clin, 2*, 836-843. doi:10.1016/j.nicl.2013.06.005

Irish, M., Hodges, J. R., & Piguet, O. (2013). Episodic future thinking is impaired in the behavioural variant of frontotemporal dementia. *Cortex, 49*(9), 2377-2388. doi:10.1016/j.cortex.2013.03.002

Irish, M., Piguet, O., Hodges, J. R., & Hornberger, M. (2014). Common and unique gray matter correlates of episodic memory dysfunction in frontotemporal dementia and Alzheimer's disease. *Hum Brain Mapp, 35*(4), 1422-1435. doi:10.1002/hbm.22263

Kipps, C. M., Nestor, P. J., Acosta-Cabronero, J., Arnold, R., & Hodges, J. R. (2009). Understanding social dysfunction in the behavioural variant of frontotemporal dementia: the role of emotion and sarcasm processing. *Brain, 132*(Pt 3), 592-603. doi:10.1093/brain/awn314

Lagarde, J., Valabregue, R., Corvol, J. C., Garcin, B., Volle, E., Le Ber, I., . . . Levy, R. (2015). Why do patients with neurodegenerative frontal syndrome fail to answer: 'In what way are an orange and a banana alike?'. *Brain, 138*(Pt 2), 456-471. doi:10.1093/brain/awu359

Lagarde, J., Valabregue, R., Corvol, J. C., Pineau, F., Le Ber, I., Vidailhet, M., . . . Levy, R. (2013). Are frontal cognitive and atrophy patterns different in PSP and bvFTD? A comparative neuropsychological and VBM study. *PLoS One, 8*(11), e80353. doi:10.1371/journal.pone.0080353

Lee, S. E., Sias, A. C., Mandelli, M. L., Brown, J. A., Brown, A. B., Khazenzon, A. M., . . . Seeley, W. W. (2017). Network degeneration and dysfunction in presymptomatic C9ORF72 expansion carriers. *Neuroimage Clin, 14*, 286-297. doi:10.1016/j.nicl.2016.12.006

Libon, D. J., McMillan, C., Gunawardena, D., Powers, C., Massimo, L., Khan, A., . . . Grossman, M. (2009). Neurocognitive contributions to verbal fluency deficits in frontotemporal lobar degeneration. *Neurology, 73*(7), 535-542. doi:10.1212/WNL.0b013e3181b2a4f5

Luo, C., Hu, N., Xiao, Y., Zhang, W., Gong, Q., & Lui, S. (2020). Comparison of Gray Matter Atrophy in Behavioral Variant Frontal Temporal Dementia and Amyotrophic Lateral Sclerosis: A Coordinate-Based Meta-Analysis. *Front Aging Neurosci, 12*, 14. doi:10.3389/fnagi.2020.00014

Mandelli, M. L., Vitali, P., Santos, M., Henry, M., Gola, K., Rosenberg, L., . . . Gorno-Tempini, M. L. (2016). Two insular regions are differentially involved in behavioral variant FTD and nonfluent/agrammatic variant PPA. *Cortex, 74*, 149-157. doi:10.1016/j.cortex.2015.10.012

Muller, V. I., Cieslik, E. C., Laird, A. R., Fox, P. T., Radua, J., Mataix-Cols, D., . . . Eickhoff, S. B. (2018). Ten simple rules for neuroimaging meta-analysis. *Neurosci Biobehav Rev, 84*, 151-161. doi:10.1016/j.neubiorev.2017.11.012

Ossenkoppele, R., Pijnenburg, Y. A., Perry, D. C., Cohn-Sheehy, B. I., Scheltens, N. M., Vogel, J. W., . . . Rabinovici, G. D. (2015). The behavioural/dysexecutive variant of Alzheimer's disease: clinical, neuroimaging and pathological features. *Brain, 138*(Pt 9), 2732-2749. doi:10.1093/brain/awv191

Pardini, M., Huey, E. D., Cavanagh, A. L., & Grafman, J. (2009). Olfactory function in corticobasal syndrome and frontotemporal dementia. *Arch Neurol, 66*(1), 92-96. doi:10.1001/archneurol.2008.521

Rankin, K. P., Mayo, M. C., Seeley, W. W., Lee, S., Rabinovici, G., Gorno-Tempini, M. L., . . . Miller, B. L. (2011). Behavioral variant frontotemporal dementia with corticobasal degeneration pathology: phenotypic comparison to bvFTD with Pick's disease. *J Mol Neurosci, 45*(3), 594-608. doi:10.1007/s12031-011-9615-2

Rosen, H. J., Gorno-Tempini, M. L., Goldman, W. P., Perry, R. J., Schuff, N., Weiner, M., . . . Miller, B. L. (2002). Patterns of brain atrophy in frontotemporal dementia and semantic dementia. *Neurology, 58*(2), 198-208. doi:10.1212/wnl.58.2.198

Seeley, W. W., Crawford, R., Rascovsky, K., Kramer, J. H., Weiner, M., Miller, B. L., & Gorno-Tempini, M. L. (2008). Frontal paralimbic network atrophy in very mild behavioral variant frontotemporal dementia. *Arch Neurol, 65*(2), 249-255. doi:10.1001/archneurol.2007.38

Whitwell, J. L., Jack, C. R., Jr., Parisi, J. E., Knopman, D. S., Boeve, B. F., Petersen, R. C., . . . Josephs, K. A. (2011). Imaging signatures of molecular pathology in behavioral variant frontotemporal dementia. *J Mol Neurosci, 45*(3), 372-378. doi:10.1007/s12031-011-9533-3

Wong, S., Bertoux, M., Savage, G., Hodges, J. R., Piguet, O., & Hornberger, M. (2016). Comparison of Prefrontal Atrophy and Episodic Memory Performance in Dysexecutive Alzheimer's Disease and Behavioral-Variant Frontotemporal Dementia. *J Alzheimers Dis, 51*(3), 889-903. doi:10.3233/JAD-151016
