# Supplementary material for: Idiopathic and acquired pedophilia as two distinct disorders: an insight from neuroimaging
Source: Brain Imaging Behav. 2021 Jan 28;15(5):2681–92. doi: 10.1007/s11682-020-00442-z (PMC8500885; doi:10.1007/s11682-020-00442-z)
Supplement: Supplementary file 2 — (PDF 370 KB) [file 11682_2020_442_MOESM2_ESM.pdf]

Reference	number bvFTD	Contrast	Coordinate system	Coordinates
Rosen 2002	8	bvFTD vs Controls and DS	Talairach	38 18 -6
				-38 26 -9
				48 17 32
				-6 50 23
				2 57 -2
				-36 0 57
Seeley 2008	15	Mild bvFTD vs controls	MNI	2 36 14
nb three independent groups				-2 36 12
				2 44 20
				-2 48 22
				2 53 -4
				-2 54 -4
				2 8 -4
				-4 12 -6
				42 17 -10
				-39 17 -13
				39 23 5
				-34 24 4
				33 36 -13
				-29 38 -13
				28 61 -15
				-31 61 -2
				30 52 18
				-42 52 9
				38 14 33
				-44 13 28
				47 -6 33
				-53 -30 -8
				45 36 17
				29 -13 -13

				-7 15 6
				9 13 9
				30 4 8
				-22 3 6
				2 -8 10
Seeley 2008	15	Moderate bvFTD vs controls	MNI	4 38 20
nb three independent groups				-2 38 16
				2 44 30
				-2 46 28
				-2 58 2
				-2 54 -2
				4 8 -8
				-4 8 -6
				36 18 -8
				-38 20 -10
				35 18 5
				-32 22 4
				34 36 -13
				-29 38 -14
				24 61 -13
				-29 61 -2
				38 52 6
				-42 53 7
				38 15 33
				-44 11 27
				45 34 20
				-48 36 26
				26 34 40
				-40 25 46
				0 36 38
				-24 19 47
				48 -18 -4

				30 -14 -10
				-8 13 9
				9 13 9
				27 6 8
				-22 5 4
				2 -8 8
Seeley 2008	15	Severe bvFTD vs controls	MNI	2 28 18
nb three independent groups				-2 32 16
				2 44 28
				-2 46 24
				1 53 -3
				-2 54 -4
				4 8 -6
				-4 10 -6
				42 16 -12
				-39 17 -13
				35 16 7
				-36 24 4
				42 16 -12
				-30 38 -15
				44 -10 -4
				-40 -12 -2
				33 63 -6
				-27 61 -3
				36 44 28
				-45 53 4
				38 13 34
				-43 10 25
				47 -6 34
				-54 -29 -4
				44 34 24
				-56 28 18

				30 26 40
				-42 28 45
				0 36 38
				-25 21 47
				50 -18 -6
				-50 -51 26
				48 -59 44
				-30 -14 -10
				28 -14 -16
				27 -26 -10
				-28 -32 -6
				-10 15 7
				9 12 7
				29 8 10
				-22 11 4
				2 -8 8
				48 -67 -48
Pardini 2009	22	bvFDT vs Controls	MNI	58 40 22
				34 28 58
				4 28 64
				8 12 14
				-4 0 14
Libon 2009	51	bvFDT vs Controls	MNI	-44 56 -8
				-30 40 18
				-30 -16 65
				-24 18 -19
				-42 10 12
				-50 -7 -25
				-36 -52 41
				-30 -85 19
				-42 -70 -2
				40 48 -2

				20 35 30
				18 33 33
				40 -2 -36
Kipps 2009	21	bvFTD vs controls	MNI	40 0 -8
				34 20 -26
				26 16 -20
				-36 16 -6
				-30 14 -16
				-50 16 -14
				6 -54 68
				60 18 18
				6 4 8
				10 16 8
				70 -42 20
				-24 -8 -40
				-24 -2 -46
Agosta 2009	31	bvFTD vs controls	MNI	48 32 28
				-48 44 -6
				8 14 50
				38 20 4
				-36 16 6
				18 22 56
				-20 52 30
				40 40 32
				-44 48 12
				30 60 -4
				-28 62 -4
				-48 44 -6
				6 44 24
				-2 36 36
				8 24 42
				68 -38 -28

Whitwell 2011	15	Pick vs Controls	MNI	-33 18 3
				-27 51 19
				-23 60 -4
				-3 39 42
				33 19 -14
				59 12 6
				4 40 36
				9 64 15
				6 33 -28
				-27 38 37
				-45 29 -15
Rankin 2011	5	Pick vs Controls	MNI	36 20 -10
				12 16 2
				22 46 38
				6 38 -26
				54 32 18
				48 22 28
				42 10 56
				70 -22 -22
				54 -48 -24
				64 -4 -30
				64 -38 46
				48 -70 -20
				8 -92 -22
				-36 14 -10
				-8 14 2
				-28 12 60
				-42 16 52
				-48 14 24
				-64 -38 38
				-54 -16 56
				-60 -66 -22

				-46 -52 -56
Hornberger 2011	14	bvFTD vs controls	MNI	-4 24 -16
				-14 8 54
				-20 16 54
Irish 2013 Cortex	10	bvFDT vs Controls	MNI	-10 28 -26
				32 -6 -50
				-40 -14 -48
				6 0 4
				-18 -14 -26
				-32 22 4
				50 12 -4
				48 -20 10
				-18 26 42
				-14 22 0
				-14 60 -12
				32 -12 -24
				-36 22 8
				-44 -58 14
				-20 10 44
				22 12 -10
Lagarde 2013	16	bvFTD vs controls	MNI	-4 40 -27
				28 8 -26
				4 32 45
				36 -9 -18
				15 60 16
				-54 8 -24
				-2 17 34
				32 54 9
Irish 2013 NeuroImage Clinical	8	C9 bvFTD vs controls	MNI	-52 6 -8
				40 -24 18
				28 -24 54

				-16 -24 60
				10 -100 22
				-32 -68 28
				-34 4 38
				-14 -86 2
				10 -18 42
				-14 -20 48
				46 -8 26
Irish 2013 NeuroImage Clinical	15	sporadic bvFTD vs controls	MNI	48 -48 54
Irish 2014 HBM	19	bvFTD vs controls	MNI	34 -8 -52
				24 -100 -14
				50 -40 -22
				-16 -12 -12
				12 -66 64
				24 -34 -6
				-54 -22 -16
				44 -52 8
				20 -54 10
				38 -72 44
				52 -46 28
				-38 -66 14
Baez 2015	16	bvFDT vs Controls	MNI	38 -20 -30
				24 -6 -16
				-13 -36 3
				-24 9 -20
				48 10 -20
				66 -45 10
				46 -79 7
				-4 -1 57
				6 0 43
				-4 -34 49
				-42 -4 54

				48 33 9
				-23 -78 43
				-24 -84 30
				60 -9 4
				-13 -72 12
				13 -57 28
				15 -60 19
				1 37 -12
Ossenkoppele 2015	59	bvFTD vs controls	not reported	24 8 -9
			MNI	-58 -24 -8
				12 47 40
				9 35 52
				-10 48 9
				-9 -16 4
				-42 3 46
				43 11 27
				37 32 7
				-10 45 42
				-22 56 21
				-45 44 -2
				30 45 -14
				54 15 13
				18 36 43
				-54 6 -27
Lagarde 2015	18	bvFTD vs controls	MNI	3 50 19
				4 32 45
				-30 23 -11
				-3 40 -26
				26 9 -32
				3 60 4
				-2 17 34
				-30 17 -30

Bohour 2016	15	bvFTD vs controls	MNI	47 -17 -8
				-39 -2 1
				-8 13 10
				9 16 8
				18 -28 13
				26 62 22
				-16 69 0
				5 49 -27
				49 31 -16
				5 36 21
				-3 26 45
				20 -25 41
				66 -29 41
				37 -75 46
				68 -30 16
				-58 -34 -7
				-1 44 -26
				-3 -10 57
				64 -49 33
				-2 52 7
Wong 2016	22	bvFTD vs controls	MNI	-36 -6 -50
				-18 18 46
				-40 18 20
				36 8 26
				22 -12 48
				-36 -32 -8
Mandelli 2016	13	bvFTD vs controls	MNI	2 33 -12
				-29 35 -15
				18 23 61
				35 11 -36
				54 -12 -23
				3 45 10

				-6 45 10
				39 21 0
				-33 14 -12
				11 9 9
				-9 11 9
				14 6 -3
				-21 -1 7
				-26 6 9
Lee 2017	15	presymptomatic bvFTD vs controls	MNI	36 51 24
				-2 -10 39
				-42 15 30
				-14 -32 4
Bertoux 2018	35	bvFTD vs controls	MNI	14 -18 0
				26 -74 -56
				-21 34 43
				3 25 43
				-42 7 28
				41 7 28
				-16 -19 10
				23 -24 10
				-10 29 -19
Hua 2018	23	bvFTD vs controls	MNI	-2 -18 10
				-14 -14 20
				12 14 15
				38 10 6
				39 20 3
				44 0 -9
				64 -2 -14
				60 2 -24
				-3 28 34
				0 36 10

				0 36 20
				54 38 -2
				46 22 15
				45 52 -12
				-51 9 -36
				-60 2 -26
				-22 34 36
				-20 34 36
				38 0 -48
				-27 51 16
				-30 60 2
				-40 44 24
				-34 18 4
				-42 15 -12
				-32 8 -21
				-36 21 52
				-2 14 56
				-69 -30 -16
				-69 -30 -16
				48 36 27
Baez 2016	26	bvFDT vs Controls	MNI	28 4 -16
				34 -7 -24
				28 1 -30
				10 19 -22
				13 46 -3
				4 43 12
				-45 -16 -27
				-36 -3 -18
