# Supplementary material for: Idiopathic and acquired pedophilia as two distinct disorders: an insight from neuroimaging
Source: Brain Imaging Behav. 2021 Jan 28;15(5):2681–92. doi: 10.1007/s11682-020-00442-z (PMC8500885; doi:10.1007/s11682-020-00442-z)
Supplement: Supplementary file 3 — (DOC 92.5 KB) [file 11682_2020_442_MOESM3_ESM.doc]

**Idiopatic Pedophilia meta-analysis**

**
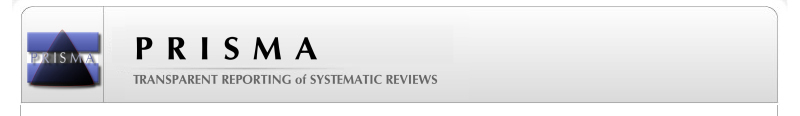
**

**PRISMA 2009 Flow Diagram**

Records identified through other databases (ETHOS, Kings Open Portal, EMBASE, MEDLINE, PsycINFO and CINHAL)
(n = 32)

**Screening**

**Included**

**Eligibility**

**Identification**

Records identified through

Pubmed searching
(n = 180)

Records after duplicates removed
(n = 212)

Records screened
(n = 212)

Records excluded

(not primary studies, no full text retrievable, no neuroimaging, participants were victims rather than offenders)
(n = 185)

Full-text articles assessed for eligibility (n = 27)

Full-text articles excluded, (see supplementary material for reasons)
(n = 8)

Comparisons included in quantitative synthesis

(meta-analysis)
(n = **19**)

**Table of Studies Excluded from the Idiopatic Pedophilia meta-analysis**

| **Reference** | **Reason for exclusion** |
| --- | --- |
| (Cohen et al., 2002) | PET study reporting ROI analysis only. In addition, for significant results, coordinates are not reported |
| (Schiltz et al., 2007) | ROI study |
| (Cantor et al., 2008) | No coordinates reported (volumetry study) |
| (Habermeyer et al., 2012) | Single case study |
| (Lett et al., 2018) | Coordinates not reported in the main paper neither in the supplementary material |
| (Ristow et al., 2018) | ROI study |
| (Gibbels et al., 2019) | The comparison presented is within convicted and not convicted pedophiles who committed sexual offenses, thus all the included participants are pedophiles and all of them committed sexual offenses |
| (Cazala et al., 2019) | No coordinates reported for the contrast of interest (contrast 5 in the paper: patients vs controls). No significant results for the second contrast of interest (contrast 6 in the paper: offenders vs not offenders) |

Cantor, J. M., Kabani, N., Christensen, B. K., Zipursky, R. B., Barbaree, H. E., Dickey, R., . . . Blanchard, R. (2008). Cerebral white matter deficiencies in pedophilic men. *J Psychiatr Res, 42*(3), 167-183. doi:10.1016/j.jpsychires.2007.10.013

Cazala, F., Fonteille, V., Moulier, V., Pelegrini-Issac, M., De Beaurepaire, C., Abondo, M., . . . Stoleru, S. (2019). Brain responses to pictures of children in men with pedophilic disorder: a functional magnetic resonance imaging study. *Eur Arch Psychiatry Clin Neurosci, 269*(6), 713-729. doi:10.1007/s00406-018-0933-z

Cohen, L. J., Nikiforov, K., Gans, S., Poznansky, O., McGeoch, P., Weaver, C., . . . Galynker, I. (2002). Heterosexual male perpetrators of childhood sexual abuse: a preliminary neuropsychiatric model. *Psychiatr Q, 73*(4), 313-336. doi:10.1023/a:1020416101092

Gibbels, C., Sinke, C., Kneer, J., Amelung, T., Mohnke, S., Beier, K. M., . . . Kruger, T. H. C. (2019). Two Sides of One Coin: A Comparison of Clinical and Neurobiological Characteristics of Convicted and Non-Convicted Pedophilic Child Sexual Offenders. *J Clin Med, 8*(7). doi:10.3390/jcm8070947

Habermeyer, B., Handel, N., Lemoine, P., Klarhofer, M., Seifritz, E., Dittmann, V., & Graf, M. (2012). LH-RH agonists modulate amygdala response to visual sexual stimulation: a single case fMRI study in pedophilia. *Neurocase, 18*(6), 489-495. doi:10.1080/13554794.2011.627346

Lett, T. A., Mohnke, S., Amelung, T., Brandl, E. J., Schiltz, K., Pohl, A., . . . Walter, H. (2018). Multimodal neuroimaging measures and intelligence influence pedophile child sexual offense behavior. *Eur Neuropsychopharmacol, 28*(7), 818-827. doi:10.1016/j.euroneuro.2018.05.002

Ristow, I., Li, M., Colic, L., Marr, V., Fodisch, C., von During, F., . . . Walter, M. (2018). Pedophilic sex offenders are characterised by reduced GABA concentration in dorsal anterior cingulate cortex. *Neuroimage Clin, 18*, 335-341. doi:10.1016/j.nicl.2018.01.018

Schiltz, K., Witzel, J., Northoff, G., Zierhut, K., Gubka, U., Fellmann, H., . . . Bogerts, B. (2007). Brain pathology in pedophilic offenders: evidence of volume reduction in the right amygdala and related diencephalic structures. *Arch Gen Psychiatry, 64*(6), 737-746. doi:10.1001/archpsyc.64.6.737
