# Supplementary material for: Idiopathic and acquired pedophilia as two distinct disorders: an insight from neuroimaging
Source: Brain Imaging Behav. 2021 Jan 28;15(5):2681–92. doi: 10.1007/s11682-020-00442-z (PMC8500885; doi:10.1007/s11682-020-00442-z)
Supplement: Supplementary file 4 — (PDF 486 KB) [file 11682_2020_442_MOESM4_ESM.pdf]

Reference	Number of Pedophiles	Number of controls	Age pedophiles	Age controls	Coordinate system	Coordinates
Schiffer et al., 2007. *"Structural brain abnormalities in the frontostriatal system and cerebellum in pedophilia"*	18	24	37.67 ± 7.99	33.63 ± 7.07	MNI	30 -71-53<br>-33 -45 -39<br>-43 -50 -43<br>-15 22 -20<br>15 26 -19<br>11 -30 45<br>-11 -36 42<br>11 -42 45<br>-14 -65 12<br>-35 0 12<br>38 -10 13<br>-10-74 20<br>13 -73 20<br>33 -36 -12<br>-32 -32 -14<br>-50 -28 6<br>51 -51 11<br>40 9 -45<br>26 0 7<br>-22 6 5
Walter et al., 2007. *"Pedophilia is Linked to Reduced Activation in Hypothalamus and Lateral Prefrontal Cortex During Visual Erotic Stimulatio"*	13	13	NA	NA	MNI	-3 -3-15<br>3 -30 -18<br>-27 42 36<br>60 -51 6<br>57 27 0<br>24-78 6<br>-30 57 24<br>30 39 51<br>42 -3 42

						-21 -66 15<br>-33 12-3<br>-6 66 24<br>12 -45 3<br>-21 -21 -15<br>24 -60 60
Sartorius et al., 2008. *"Abnormal amygdala activation profile in pedophilia"*	10	10	33.1 ± 8.9	35.3 ± 8.3	MNI	-27 -3 -24<br>30 3 -24
Schiffer et al., 2008. *"Functional brain correlates of heterosexual paedophilia"*	8	12	38.4 ± 9.4	36.1 ± 7.5	MNI	-9 12 33<br>3 36 9<br>3 15 27<br>6 21 21<br>-6 -54 3<br>-48 15 -9<br>54 -3 -12<br>21 -81 30<br>9 -75 30<br>- 9 -66 30<br>-12 -57 30<br>-6 -63 0<br>24 -78 -18<br>-27 -36 -6<br>-33 -27 -12<br>30 24 -9<br>-36 -39 -12<br>51 -60-21<br>18 -24 15<br>30 21 -24<br>-30 15 -30<br>45 33 -12<br>6 57 3

							3 -15 39<br>6 3 42<br>-3 6 39<br>-3 38 0<br>3 42 3<br>42 -18 63<br>-30 -15 69<br>-3 -24 48<br>48 18 27
Schiffer et al., 2008. *"Brain response to visual sexual stimuli in homosexual pedophiles"*	11		12	37.0 ± 7.5)	32.0 ± 6.8	MNI	6 30 0<br>3 30 -9<br>-6 39 3<br>3 54 0<br>12 18 15<br>9 9 18<br>30 -33 -21<br>-36 36 15<br>-42 33 21
Poeppl et al., 2011.*"Functional Cortical and Subcortical Abnormalities in Pedophilia: A Combined Study Using a Choice Reaction Time Task and fMRI"*	2	7	11	45 ± 8	29 ± 6	MNI	44-40 -2<br>12 -42 30<br>-8 -16 14<br>22 12 26<br>34 -20 -16<br>42 -4 -24<br>28 -44 -24<br>32 12 16<br>48 -22 48<br>8 -12 36<br>-40 -44 22

										42 8 16<br>46 -42 -2
Ponseti et al., 2012. *"Assessment of Pedophilia Using Hemodynamic Brain Response to Sexual Stimuli"*	11	#	18	#	37.0 ± 5.9	33.5 ± 14.2	32.4 ± 8.2	28.6 ± 5.7	MNI	−38 −62 −24<br>40 −64 −22<br>−12 −40 −2<br>−2 −6 4<br>22 −32 −4<br>−34 −76 0<br>14 −34 0<br>−38 −74 −16<br>46 −44 −12<br>38 −66 44<br>12 18 −8<br>−8 14 −8<br>32 −62 52<br>−42 −44 −10<br>−38 −46 −10<br>−2 4 28<br>−38 −78 0<br>−18 0 −12<br>−26 −56 46<br>44 −42 −14<br>54 −56 −8<br>−32 12 −10<br>44 24 22<br>−16 −8 10<br>28 −48 −30
Habermayer et al., 2013. *"Response inhibition in pedophilia: an FMRI pilot study"*	11		7		49 ± 12.5		47 ± 8.6		Tailarach	−40 −62 48

						−46 −65 30<br>−52 −47 30<br>−7 −35 36
Habermayer et al., 2013. *"Immediate processing of erotic stimuli in paedophilia and controls: a case control study"*	8	8	48.25 ± 9.15	46.25 ± 8.38	Tailarach	51 −34 −5<br>45 −17 −12<br>41 −42 34<br>41 −78 −11<br>32 −85 8<br>9 −94 −8<br>6 83 −29<br>−33 −90 −15<br>−20 −86 −20<br>−25 −85 −32<br>11 57 28<br>29 54 4
Poeppl et al., 2013. *"Association between brain structure and phenotypic characteristics in pedophilia"*	9	11	45 ± 8	29 ± 6	MNI	30 -12 12<br>-44 -22 18<br>-36 29 9<br>3 26 -29<br>-40 -58 21<br>36 -57 19
Cantor et al., 2015. *"Diffusion Tensor Imaging of Pedophilia"*	24	32	35.63 ± 9.52	37.00 ± 10.72	MNI	-36 -86 -8<br>-72 -20 10<br>10 -96 28

						72 -8 -2<br>-22 -78 -56<br>38 -74 -60<br>20 -30 -42<br>8 -6 -32<br>56 -12 60<br>-6 44 -32<br>72 -40 -18<br>-6 18 74<br>28 -80 0<br>-36 -42 -60<br>36 66 -6<br>-36 -78 4<br>-68 -20 -28<br>-48 4 -48<br>-68 -22 42<br>-32 60 -20<br>46 16 -44<br>-50 -76 30<br>16 -66 72<br>-42 -46 62<br>-54 -14 58<br>64 8 -10<br>36 -34 74<br>26 64 24<br>-32 42 48<br>72 -30 34

Gerwinn et al., 2015. *"The (in)consistency of changes in brain macrostructure in male paedophiles: A combined T1-weighted and diffusion tensor imaging study"*	11	#	18 #	/	/	MNI	38 -52 52<br>47 -37 49<br>44 -45 54
Kargel et al., 2015. *"Diminished Functional Connectivity on the Road to Child Sexual Abuse in Pedophilia"*	4 (CSA-)	10 (CSA-)	14	28.07 ± 5.71, 20–40	32.86 ± 9.89, 21–49	MNI	-57 28 6<br>-15 -102 20<br>-19 21 34
	4 (CSA+)	8 (CSA+)	14	43.67 ± 7.08, 29–55	32.86 ± 9.89, 21–49	MNI	-15 32 -12<br>-57 -25 -36<br>-47 -77 -29<br>-50 -18 -1<br>73 -21 10<br>-26 -25 59<br>-22 70 3<br>-26 -91 -19<br>-5 49 -19<br>66 -53 3<br>-1 -28 38<br>-15 -11 24<br>-61 -67 3
Cantor et al., 2016. *"Independent Component Analysis of Resting-State Functional Magnetic Resonance Imaging in Pedophiles"*	37		67	35.70	35.95/ 40.50	MNI	6 -14 16<br>38 -86 8<br>54 34 20<br>-34 -90 4<br>38 -54 44<br>-38 -14 4<br>6 -46 -48<br>6 -46 -20

						38 -18 28<br>6 -58 -68<br>18 -62 -56<br>-2 -22 -28<br>46 -42 12<br>42 -90 -12<br>50 -18 16<br>-14 -102 8<br>30 -50 -8<br>50 -66 0<br>-2 -18 44<br>18 -62 -12<br>-46 22 24<br>22 -102 12<br>-34 -22 16
Kargel 2017	40 Cso+ 37 CSO-				MNI	-7 -45 25<br>-2 -61 28<br>-20 24 43
Massau et al., 2017. *"Neural correlates of moral judgment in pedophilia"*	31	19	36.44 ± 8.01/31.73 ± 6.49	33.47 ± 10.24	MNI	-20 -74 14<br>-38 -42 14<br>-14 -16 32<br>12 -30 46
Shiffer 2017<br>Gray matter anomalies in pedophiles with and without a history of child sexual offending	58 CSO+ 60 CS=-	101 HC			MNI	-44 -51 -26<br>8 -12 43<br>-4 39 -12<br>-42 2 -12<br>-14 35 -27<br>-27 -33 -24<br>-4 41 15<br>-28 8 -45

Ponseti et al., 2017. *"Decoding Pedophilia: Increased Anterior Insula Response to Infant Animal Pictures"*	60	55	36.6 ± 10.7	35 ± 10.2	MNI	36 26 4<br>28 56 24<br>34 34 18<br>12 2 54<br>10 16 54<br>-4 6 56<br>-28 34 20<br>-28 46 16<br>-18 54 16<br>58 -34 -16
Fonteille 2019	#	#	42	41	MNI	8 50 -24
