# Supplementary material for: Idiopathic and acquired pedophilia as two distinct disorders: an insight from neuroimaging
Source: Brain Imaging Behav. 2021 Jan 28;15(5):2681–92. doi: 10.1007/s11682-020-00442-z (PMC8500885; doi:10.1007/s11682-020-00442-z)
Supplement: Supplementary file 5 — (DOC 47.0 KB) [file 11682_2020_442_MOESM5_ESM.doc]

**Table of Excluded Cases Reports of Acquired Pedophilia**

| **Reference** | **Reason for exclusion** |
| --- | --- |
| (von Krafft-Ebing, 1897) | First case described in the literature of acquired pedophilia. Excluded from the current analysis as the dementia the patient is suffering with is not specified |
| (Regestein & Reich, 1978) | Cases 2,3,4 Excluded as the etiology and acquired origin of pedophilia was not clear. |
| (Solla, Floris, Tacconi, & Cannas, 2006) | Excluded as the patient manifested pedophilic urges caused by a self-administration of excessive extra doses of dopaminergic drugs to treat parkinsonism. Thus, a clear structural neural basis is absent. |
| (Prahlada Rao, Chand, & Murthy, 2007) | Excluded as the etiology of pedophilic insurgence was not clear and the MRI showed no abnormalities in the brain. |
| (Mendez & Shapira, 2011) Case 3 | This patient manifested pedophilia probably as a result of Alzehimer’s disease, frontal lobe variant. However, the MRI revealed atrophy to the temporal lobes. Due to this inconsistency, this patient was excluded. |
| (Mendez & Shapira, 2011) Case 5 | This patient was excluded as he manifested pedophilia as a symptom of addition to pramipaxone, to treat Parkinson’s disease. Thus, a clear structural neural basis is absent. |
| (Farisco & Petrini, 2014) | This paper is commenting upon a case previously described (Sartori, Scarpazza, Codognotto, & Pietrini, 2016; Scarpazza, Pellegrini, Pietrini, & Sartori, 2018) |

Farisco, M., & Petrini, C. (2014). On the stand. Another episode of neuroscience and law discussion from Italy. *Neuroethics, 7*, 243-245.

Mendez, M., & Shapira, J. S. (2011). Pedophilic behavior from brain disease. *J Sex Med, 8*(4), 1092-1100. doi:10.1111/j.1743-6109.2010.02172.x

Prahlada Rao, N., Chand, P. K., & Murthy, P. (2007). A case of late-onset pedophilia and response to sertraline. *Prim Care Companion J Clin Psychiatry, 9*(3), 235-236. doi:10.4088/pcc.v09n0311e

Regestein, Q. R., & Reich, P. (1978). Pedophilia occurring after onset of cognitive impairment. *J Nerv Ment Dis, 166*(11), 794-798.

Sartori, G., Scarpazza, C., Codognotto, S., & Pietrini, P. (2016). An unusual case of acquired pedophilic behavior following compression of orbitofrontal cortex and hypothalamus by a Clivus Chordoma. *J Neurol, 263*(7), 1454-1455. doi:10.1007/s00415-016-8143-y

Scarpazza, C., Pellegrini, S., Pietrini, P., & Sartori, G. (2018). The role of Neuroscience in the Evaluation of Mental Insanity: on the controversies in Italy. *Neuroethics, 11*(1), 83-95.

Solla, P., Floris, G., Tacconi, P., & Cannas, A. (2006). Paraphilic behaviours in a parkinsonian patient with hedonistic homeostatic dysregulation. *Int J Neuropsychopharmacol, 9*(6), 767-768. doi:10.1017/S1461145705006437

von Krafft-Ebing, B. (1897). Trattato di Psicopatologia Forense. *Fratelli Bocca, Editori (Torino)*, 215.
